# Canadian hereditary angioedema guideline

**DOI:** 10.1186/1710-1492-10-50

**Published:** 2014-10-24

**Authors:** Stephen Betschel, Jacquie Badiou, Karen Binkley, Jacques Hébert, Amin Kanani, Paul Keith, Gina Lacuesta, Bill Yang, Emel Aygören-Pürsün, Jonathan Bernstein, Konrad Bork, Teresa Caballero, Marco Cicardi, Timothy Craig, Henriette Farkas, Hilary Longhurst, Bruce Zuraw, Henrik Boysen, Rozita Borici-Mazi, Tom Bowen, Karen Dallas, John Dean, Kelly Lang-Robertson, Benoît Laramée, Eric Leith, Sean Mace, Christine McCusker, Bill Moote, Man-Chiu Poon, Bruce Ritchie, Donald Stark, Gordon Sussman, Susan Waserman

**Affiliations:** University of Toronto, Toronto, Ontario Canada; HAE Canada, Notre Dame des Lourdes, Manitoba, Canada; Department of Medicine, Laval University, Quebec City, Quebec Canada; Department of Medicine, University of British Columbia, Vancouver, British Columbia Canada; Department of Medicine, McMaster University, Hamilton, Ontario Canada; Department of Medicine, Dalhousie University, Halifax, Nova Scotia Canada; University of Ottawa Medical School, Ottawa, Ontario Canada; Goethe-Universität Frankfurt am Main, Frankfurt am Main, Germany; Department of Internal Medicine, University of Cincinnati, Cincinnati, Ohio USA; Department of Dermatology, University Hospital of the Johannes Gutenberg-University of Mainz, Mainz, Germany; Hospital La Paz Health Research Institute, Madrid, Spain; Department of Internal Medicine, UniversitadegliStudi di Milano, Ospedale L. Sacco, Milan, Italy; Departments of Medicine and Pediatrics, Penn State University, Hershey, Pennsylvania USA; 3rd Department of Internal Medicine, Faculty of Medicine, Semmelweis University, Budapest, Hungary; Department of Immunology, Barts and the London NHS Trust, London, England, UK; University of California, San Diego, San Diego, California USA; HAE International, Skanderborg, Denmark; Department of Medicine, Queen’s University, Kingston, Ontario Canada; Departments of Medicine and Paediatrics, University of Calgary, Calgary, Alberta Canada; Saskatoon Health Region, Saskatoon, Saskatchewan Canada; BC Children’s Hospital, Vancouver, British Columbia Canada; Centre hospitalier de l’université de Montréal, Montréal, Quebec Canada; Department of Medicine, University of Toronto, Oakville, Ontario Canada; Department of Immunology, McGill University Health Centre, Montreal, Quebec Canada; Department of Medicine, Western University, London, Ontario Canada; Southern Alberta Rare Blood and Bleeding Disorders Comprehensive Care Program, Calgary, Alberta Canada; Departments of Medicine and Medical Oncology, University of Alberta, Edmonton, Alberta Canada

**Keywords:** Hereditary angioedema, Guideline, Recommendations, Acute attacks, Short-term prophylaxis, Long-term prophylaxis, Self-administration, Individualized therapy, Quality of life, Comprehensive care, GRADE

## Abstract

Hereditary angioedema (HAE) is a disease which is associated with random and often unpredictable attacks of painful swelling typically affecting the extremities, bowel mucosa, genitals, face and upper airway. Attacks are associated with significant functional impairment, decreased Health Related Quality of Life, and mortality in the case of laryngeal attacks. Caring for patients with HAE can be challenging due to the complexity of this disease. The care of patients with HAE in Canada is neither optimal nor uniform across the country. It lags behind other countries where there are more organized models for HAE management, and where additional therapeutic options are licensed and available for use. The objective of this guideline is to provide graded recommendations for the management of patients in Canada with HAE. This includes the treatment of attacks, short-term prophylaxis, long-term prophylaxis, and recommendations for self-administration, individualized therapy, quality of life, and comprehensive care. It is anticipated that by providing this guideline to caregivers, policy makers, patients and their advocates, that there will be an improved understanding of the current recommendations regarding management of HAE and the factors that need to be considered when choosing therapies and treatment plans for individual patients. The primary target users of this guideline are healthcare providers who are managing patients with HAE. Other healthcare providers who may use this guideline are emergency physicians, gastroenterologists, dentists and otolaryngologists, who will encounter patients with HAE and need to be aware of this condition. Hospital administrators, insurers and policy makers may also find this guideline helpful.

**Electronic supplementary material:**

The online version of this article (doi:10.1186/1710-1492-10-50) contains supplementary material, which is available to authorized users.

## Introduction

### Background

Hereditary angioedema due to C1 inhibitor deficiency (C1-INH-HAE) is an autosomal dominant condition with an estimated prevalence of approximately 1:50,000 [1, 2]. It results in random and often unpredictable attacks of painful swelling typically affecting the extremities, bowel mucosa, genitals, face and upper airway [3]. Attacks are associated with significant functional impairment, decreased Health Related Quality of Life (HRQoL), and mortality in the case of laryngeal attacks [4, 5]. The swelling in HAE is a result of impaired regulation of bradykinin synthesis [6]. Bradykinin is a nonapeptide kinin formed from high molecular weight kininogen by the action of plasma kallikrein. Bradykinin is a very powerful vasodilator and increases capillary permeability, constricts smooth muscle and stimulates pain receptors [1].

HAE can be categorized into 3 different types depending on the level and function of C1inhibitor (C1-INH): type 1(HAE-1), type 2 (HAE-2), and HAE with normal C1-INH function (HAE-nC1INH) previously referred to as type 3 (Table 1). HAE-1 is the most prevalent, representing approximately 85% of cases and results from low antigenic and functional levels of C1-INH. HAE-2 accounts for approximately 15% of cases and is associated with a normal C1-INHprotein level but impaired C1-INH function [7, 8]. C4 is reduced in 98% of cases for both HAE-1 and HAE-2, and nearly 100% of the time during an attack [7].

**Table 1:** **Laboratory findings in hereditary angioedema**

	C4	C1-INHAntigen		C1INH Function
HAE - 1	↓	↓		↓
HAE - 2	↓	Normal or	↑	↓
HAE - nC1INH				
-FXII mutation	Normal	Normal		Normal
-UnknownCause	Normal	Normal		Normal

References [9, 10].

HAE-nC1INH (previously referred to as type 3 HAE), is much less prevalent than HAE-1 and HAE-2. The true prevalence is not known as there are no reliable assays to screen for this condition. In about 20% to 25% of identified patients, causative mutations in the gene coding for the coagulation factor XII (*F*12) have been found (HAE-nC1INH-FXII) whereas in the remaining patients no genetic cause has been identified up to now (HAE-nC1INH-unknown) [11–13]. The exact pathogenesis, however, including the mode of action of the *F*12 gene mutations and the role of estrogens is still unknown. The lack of laboratory and genetic assays (with the exception of *F*12 gene mutations) to diagnose HAE-nC1INH, has made the identification of these patients more difficult than patients with HAE-1 or HAE-2. A recent international consensus group has published criteria to make the diagnosis of HAE-nC1INH [13]. These included a history of recurrent angioedema in the absence of concomitant hives or use of medication known to cause angioedema; documented normal or near normal C4, C1-INH antigen and function; and either a *F*12gene mutation associated with the disease, or family history of angioedema and documented lack of efficacy of chronic high dose non-sedating antihistamine therapy [13].

Management of HAE can be divided into various approaches. The aim of treatment of acute attacks, also referred to as ‘on demand therapy’ is to minimize their severity, including potentially fatal upper airway edema, and associated impairment of Quality of Life (QoL). Short term prophylaxis (STP) refers to treatment meant to minimize the risk of attacks when avoidance of potential and known triggers is not possible. Long term prophylaxis (LTP) refers to ongoing treatment of HAE aimed at minimizing the overall number, frequency and/or severity of attacks. The details of specific therapies for these treatment approaches will be discussed in the sections that follow. In addition to the evidence behind the proposed recommendations, the clinical considerations for their implementation will also be discussed. The United States Preventive Services Task Force describes clinical considerations as statements that can help clinicians by offering practical information so they can tailor guideline recommendations to individual patients [14]. This Clinical Consideration section following each recommendation is intended to help place the recommendation into a clinical context.

#### Scope and purpose

The objective of this guideline is to provide graded recommendations for the management of patients in Canada with HAE-1, HAE-2 and HAE-nC1INH. This includes the treatment of attacks, STP, LTP, and recommendations for self-administration, individualized therapy, QoL, and comprehensive care.

The care of patients with HAE in Canada is neither optimal nor uniform across the country. It lags behind other countries where there are more organized models for HAE management, and where additional therapeutic options are licensed and available for use [15]. It is anticipated that by providing this guideline to caregivers, policy makers, patients and their advocates, that there will be an improved understanding of the current recommendations regarding management of HAE and the factors that need to be considered when choosing therapies and treatment plans for individual patients.

It is not the intent of this guideline to provide a detailed description of the pathophysiology or nomenclature of HAE which can be found elsewhere [13]. It is also not intended to be prescriptive in its recommendations, but rather to highlight issues that need to be considered when choosing treatments options for patients with a focus on the importance of individualized care.

#### Intended audience

The primary target users of this guideline are healthcare providers who are managing patients with HAE-1, HAE-2 and HAE-nC1INH. Other healthcare providers who may use this guideline are emergency physicians, gastroenterologists, dentists and otolaryngologists, who will encounter patients with HAE and need to be aware of this condition. Hospital administrators, insurers and policy makers may also find this guideline helpful.

## Methods

### Committee members and consensus conference participants

The Canadian Hereditary Angioedema Guideline Committee is a working committee under the umbrella of the Canadian Hereditary Angioedema Network (CHAEN)/Réseau Canadien d'angioédème héréditaire (RCAH) http://chaen-rcah.ca/. Members on this committee included members from CHAEN/RCAH across Canada as well as the President of the Canadian HAE Patient Organization – Hereditary Angioedema (HAE) Canada/Angioédème Héréditaire (AEH) Canada. The Canadian Hereditary Angioedema Committee was responsible for defining the scope and purpose of the guideline and choosing the international participants. International participants were selected based on their contributions to the HAE literature, relating to HAE and its management, and their expertise in priority areas for this guideline including self-administration, individualized therapy, HRQoL, and comprehensive care. Those identified experts were asked to present a summary of the evidence related to these areas to all conference participants.

Conference participants included the CHAEN/RCAH Guideline Committee, international experts, all currently registered members of CHAEN who were able to attend the meeting, the President of HAE/AEH Canada and their designates, President of the international HAE patient group HAEi, Hema-Quebec, and industry representatives. An invitation was extended to representatives of the Provincial/Territorial Blood Coordinating offices.

Representatives from Industry, who manufacture products for the treatment of HAE, were also invited to provide information on their products if required during the meeting. Only medical personnel and general managers were invited but were not present during times when decisions were made. Marketing representatives were excluded.

### Funding and support

Funding for the CHAEN/RCAH Guideline Conference was done through the CHAEN/RCAH. This organization received equal support from 3 companies, who manufacture products for the treatment of hereditary angioedema (CSL Behring, Shire, and ViroPharma - Viropharma was acquired by Shire between the time of the guideline meeting and publication of the Guideline). Requests to procure funding from Provincial and National Government and Blood Agencies were not successful. Funding was used to support rental of the conference facilities, audio-taping, facilitation by an external facilitator, travel to the meeting, accommodation, and foods for all participants except for government agency representatives and patient representatives who were supported by their own agencies. No industry participants were funded by CHAEN/RCAH. No participants at the meeting were compensated for their time except for the Guideline meeting methodologist and facilitator from the Centre of Effective Practice.

The guideline meeting process was aided by a methodologist and a guideline facilitator from the Centre for Effective Practice and supported by HAE/AEH Canada.

### Conflict of interest

Details of potential conflicts of interest were elicited using the standardized International Committee of Medical Journal Editors Form for Disclosure of Potential Conflicts of Interest (Additional file 1). COI forms were distributed to attendees prior to their reviewing the manuscript, and were mandatory for all contributing authors.

### Identifying the evidence

A systematic search of Ovid MEDLINE was conducted by a librarian from the Centre for Effective Practice (KLR) on October 10, 2013, in order to identify clinical trials addressing long-and short-term prophylaxis and treatment of acute attacks in patients of any age diagnosed with HAE-1, HAE-2, or HAE-nC1INH. Outcomes of interest included frequency or severity of attacks, symptom relief and QoL measures as reported or measured by the affected subject or investigator. Studies were limited to English language publications, and there were no limits on the publication date of study other than those imposed by the database (1946-October week 1, 2013). After duplicates were removed, 416 results were found, the abstracts of which were reviewed to determine if they met the inclusion criteria. If unclear from the abstract whether the paper met these criteria, the full-text document was reviewed. One hundred and thirty two results were retrieved and reviewed in full text, and from this, 11 relevant randomized control trials and 34 lower-quality comparative studies without blinding or randomization were identified and included. No studies which met the inclusion criteria were identified for HAE-nC1INH. The full search strategy is available in Additional file 2.

### Summarizing and evaluating the evidence

Key information from the included studies such as study design, number of patients, outcome measures, side effects and funding source was extracted into evidence tables for each intervention (see Additional file 3). Evidence tables were provided to the Committee Members and were available for reference at the meeting.

Criteria for determining Levels of Evidence and Strength of Recommendation were adapted from the GRADE system, [16–18] and the process was based primarily on the Journal of Clinical Epidemiology’s 2011–2013 series of articles describing the GRADE methodology. GRADE is considered “outcome centric,” and traditionally recommends a single rating for each outcome across the full body of evidence. The method applied here involved evaluating the quality of each study individually, and then looking at the studies together to assign a Level of Evidence based on the collection of studies.

Each identified randomized control trial was assessed by two reviewers (KL-R, VP) for quality using the Cochrane Risk of Bias Tool [19]. Any disagreements were resolved by a third reviewer (SB). Randomized trials were initially rated as High quality levels of evidence, with quality being downgraded for evidence of bias on the Cochrane tool and if there was evidence of inconsistency (Additional file 3: Table S1). Non-randomized, non-blinded trials were considered to be Low quality evidence.

Multiple factors were considered when assigning the Strength of Recommendation, including quality of evidence, balance between desirable and undesirable effects, values and preferences, and costs (Additional file 3: Table S2).

Additional file 3 describes additional detail how quality was assessed and the criteria used to determine the Strength of Recommendation. The quality ratings were presented at the meeting during the discussion of draft recommendations. Additional files 4 and 5 list the HAE RCT evidence tables and the lower quality comparison study evidence respectively.

### Recommendation development and approval

The Chair (SB) developed draft recommendations based on the identified literature, and presented them to the Committee Members who approved them in draft. Invited Committee Members were assigned specific topic areas and were asked to review the evidence relevant to their topic and present the body of evidence for consideration at the Guideline meeting. After the summary was presented, the Consensus Conference Participants were provided an opportunity to discuss the literature. Following this discussion, the draft recommendation was presented and the group discussed the specific wording of the recommendation before voting anonymously via electronic voting to agree or disagree with the recommendation, or abstain. If 80% consensus was not reached, there was additional group discussion, the recommendation was rephrased, and a new vote conducted. This process was conducted a maximum of 3 times. If 80% consensus was not reached, it was considered that the committee was unable to reach consensus.

Once the phrasing of a recommendation was approved by the group, the proposed Level of Evidence was presented by the methodologist guideline facilitator (High, Moderate, Low, Very Low, or Consensus). The Level of Evidence was then discussed, revised if necessary, and similarly voted on as outlined above.

The suggested Strength of Recommendation (Strong or Weak) was then presented to the group. The methodologist guideline facilitator proposed a Strength of Recommendation based on the Level of Evidence, the balance between desirable and undesirable effects, values and preferences. These factors were discussed amongst the group before voting to accept the proposed Strength of Recommendation. All votes were recorded and presented in real time with the recommendations. Table 2 is a summary of all the recommendations, the level of evidence supporting each recommendation, and the strength of each recommendation.

**Table 2:** **Summary of recommendations**

**Recommendation**	**Level of Evidence and Strength of Recommendation**
**Treatment of Acute Angioedema Attacks**	
1. Effective therapy should be used to treat acute attacks of angioedema to reduce duration and severity of attacks.	High, Strong
2. pdC1-INH is an effective therapy for the treatment of acute attacks.	High, Strong
3. Icatibant is an effective therapy for the treatment of acute attacks.	High, Strong
4. Ecallantide is an effective therapy for the treatment of acute attacks.	High, Strong
5. rhC1-INH is an effective therapy for the treatment of acute attacks.	High, Strong
6. Attenuated androgens should not be used to treat acute attacks.	Low, Strong
7. Tranexamic acid should not be used to treat acute attacks.	Low, Strong
8. Frozen plasma could be used for treatment of acute attacks if other recommended therapies are not available.	Low, Strong
9. We recommend early treatment of attacks to reduce morbidity (Level of Evidence: Moderate) and mortality (Level of Evidence: Expert Opinion).	Moderate, Strong/Expert Opinion, Strong
10. All attacks of angioedema involving the upper airway are medical emergencies and must be treated immediately. (Level of Evidence: Low) In addition, we recommend emergency department assessment. (Level of Evidence: Expert Opinion).	Low/Expert Opinion, Strong
**Acute Treatment of HAE with Normal C1-INH**	
11. There is insufficient evidence to make a recommendation for or against the use of HAE-specific therapies in the treatment of acute attacks in patients with HAE with normal C1-INH.	Very Low / Insufficient Evidence
**Short-Term Prophylaxis**	
12. Short-term prophylaxis should be considered prior to known patient-specific triggers and for any medical, surgical or dental procedures.	Low, Strong
13. HAE-specific acute treatment should be available during and after any procedure.	Low, Strong
**Long-Term Prophylaxis In HAE 1 & 2**	
14. Long-term prophylaxis may be appropriate for some patients to reduce frequency, duration and severity of attacks.	High, Strong
15. Attenuated androgens are effective for long-term prophylaxis in some patients.	Moderate, Strong
16. Plasma-derived C1-INH is effective for long-term prophylaxis in some patients.	High, Strong
17. Anti-fibrinolytics are effective for long-term prophylaxis in some patients.	Moderate, Strong
18. It is not necessary to fail other long-term prophylaxis therapies before use of C1-INH for long-term prophylaxis is considered.	Expert Opinion, Strong
19. There is insufficient evidence to make a recommendation for or against long-term prophylaxis for patients with HAE with normal C1-INH.	Very Low/Insufficient Evidence
**Self-Administration**	
20. All patients should be trained on self-administration of HAE-specific therapies if they are suitable candidates. If patients cannot self-administer therapy, provisions should be made to ensure timely access to all appropriate therapies.	Low, Strong
**Approach to Individualized Therapy**	
21. The decision to start or stop long-term prophylaxis depends on multiple factors and should be made by the patient and an HAE specialist.	Expert Opinion, Strong
**Quality of Life**	
22. Health care providers should specifically address factors known to affect quality of life with HAE patients. Management of HAE should aim to improve patients’ quality of life.	Low, Strong
**Comprehensive Care**	
23. Comprehensive care should be available for all patients with HAE.	Low, Strong

For each topic area, group discussions were captured on audiotape, and used to inform the clinical considerations for each recommendation.

To mitigate any real or perceived bias that may have influenced the outcomes, industry representatives at the meeting were asked to leave the room after the scientific presentations and were not present during any discussion either of the data, wording of the recommendations, levels of evidence, strength of recommendations, or the voting process.

Prior to the in-person meeting, the Committee Members determined that open discussion amongst conference participants regarding an approach to individualized therapy would be beneficial. For this topic, small round table discussions were facilitated prior to recommendation review and voting, and additional clinical considerations were.

## Guideline recommendations

### Treatment of acute attacks of HAE types 1 and 2

#### Background

Acute attacks of HAE may be spontaneous or precipitated by an external stimulus and range from mild to life-threatening. The decision to treat an attack depends on many variables and the severity of an attack cannot always be predicted by its earliest manifestations. The aim of treating acute attacks is to reduce the duration and severity of an attack, to minimize the impact of an attack on the functional ability of the patient, and reduce morbidity and potential mortality.

Despite the increase in available beneficial therapies, some therapies which have not been shown to be effective in trials continue to be used in acute attacks due to either historical precedent or lack of awareness.

Nine randomized trials were identified which demonstrated improvement in duration and severity of acute attacks of HAE types 1 and 2 [20–29]. The therapies studied were plasma derived C1-INH (pdC1-INH), recombinant human C1-INH (rhC1-INH), icatibant and ecallantide. Table 3 lists the specific agents, their mechanism of action, their licensed indications in Canada, the recommended dosages, and important potential adverse reactions. The quality of individual studies is described under the drug-specific recommendations which follow. Based on the rating of each study using the Cochrane Risk of Bias Tool (see Additional file 4), as well as the overall consistent effect of therapy on the relevant outcomes (reduction of duration and severity of acute attacks), and effect size, this body of evidence determined by the conference participants to be of High quality.

**Table 3:** **Therapies for HAE supported by high level of evidence**

HAE specific treatment	Product name and company	Mechanism of Action	Approved Indications In Canada	Dose	Adverse Events
C1-INH – Plasma	Berinert® (CSL)	Replaces CI-INH	Acute treatment	20 IU/Kg intravenous	Anaphylaxis/Thrombosis (rare);Transmission of infectious agents (theoretical)
	Cinryze® (Shire)	Replaces CI-INH	Long term Prophylaxis	1000 IU q3-4 days intravenous	Anaphylaxis/Thrombosis (rare);Transmission of infectious agents (theoretical)
– Recombinant	Rhucin® (Pharming)	Replaces CI-INH	Not licensed	50 U/Kg Intravenous	Anaphylaxis (rare)
Ecallantide	Kalbitor® (Dyax)	Inhibits plasma kallikrein	Not licensed	30 mg subcutaneous injection	Anaphylaxis (uncommon)
Icatibant	Firazyr® (Shire)	Blocks bradykinin 2 receptor	Acute treatment	30 mg subcutaneous injection	Pain, swelling, pruritis at injection site (common)
					Exacerbation of coronary artery disease (theoretical)

Based on the Level of Evidence, the potential severity of the outcomes and the low risk of adverse effects, the panel voted for a Strong Recommendation in favour of the use of effective therapies in the treatment of acute attacks.

***Recommendation 1***

**Effective therapy should be used to treat acute attacks of angioedema to reduce duration and severity of attacks.**

**Level of Evidence: High** (96% Agree, 4% Disagree)

**Strength of Recommendation: Strong** (100% Agree)

#### Clinical considerations

The panel emphasized the importance of changing practice towards the use of effective therapies based on evidence based data, and specifically not using therapies which were not supported by evidence, such as antihistamines, corticosteroids and epinephrine which are directed at treating histamine mediated angioedema.

***Recommendation 2***

**pdC1-INH is an effective therapy for the treatment of acute attacks.**

**Level of Evidence: High** (100% Agree)

**Strength of Recommendation: Strong** (100% Agree)

#### Clinical considerations

The pdC1-INH is a human blood product. Treatment with pdC1-INHreplaces the deficient protein in patients with HAE-1 and HAE-2.Berinert® (CSL Canada) is the only licensed product in Canada for the treatment of acute attacks of HAE-1 and HAE-2. It has been licensed since 2010, and is available throughout Canada, through Canadian Blood Services or Hema-Quebec. It can be used to treat all attacks of HAE-1 and HAE-2 in adults and children. The recommended dosage is 20 U/kg administered intravenously either by healthcare professionals or by patients and their caregivers who have been trained in its administration. It has been shown to effectively treat acute attacks in pediatric and adult patients with HAE-1 and HAE-2 [22].

Although not currently licensed in Canada for the treatment of acute attacks, Cinryze® (Shire) is licensed in Europe for treatment of acute attacks in adolescent and adult patients with HAE-1 and HAE-2at a dose of 1000 units intravenously initially and another 1000 units if no response. It has been shown to reduce the median time to onset of unequivocal relief of symptoms compared to placebo group [29].

The dose derived for treatment of acute attacks comes from clinical trials. There have been no head to head trials comparing products so it cannot be concluded that different doses of different products were equally effective. There is some evidence that efficacy is dose dependant, but this has not been confirmed with rigorous dose finding trials [30]. The pdC1-INH products are safe and well tolerated when used as indicated with no documented transmission of infectious agents.

***Recommendation 3***

**Icatibant is an effective therapy for the treatment of acute attacks.**

**Level of Evidence: High** (97% Agree, 3% Disagree)

**Strength of Recommendation: Strong** (96% Agree/4% Disagree)

#### Clinical considerations

Bradykinin is a key mediator in inducing angioedema through activation of the bradykinin B2 receptor [6]. Icatibant is a synthetic 10-amino acid peptide and acts as a selective bradykinin B2 receptor antagonist. It is administered as a single 30 mg subcutaneous injection. It has been shown to effectively treat acute attacks in adult patients with HAE-1 and HAE-2 [20, 25]. Icatibant is licensed in Europe and the USA for self-administration for the treatment of HAE attacks. (Addendum: Icatibant was licensed by Health Canada July 16, 2014). It is generally well-tolerated, although 90% of patients experience transient local pain, swelling, and erythema at the injection site.

***Recommendation 4***

**Ecallantide is an effective therapy for the treatment of acute attacks.**

**Level of Evidence: High** (94% Agree, 6% Disagree)

**Strength of Recommendation: Strong** (94% Agree, 6% Disagree)

#### Clinical considerations

Plasma kallikrein generates bradykinin through cleavage of high-molecular-weight kininogen [6, 31, 32]. Ecallantide is a 60-amino acid recombinant protein that acts as an inhibitor of kallikrein. It is administered as three 10 mg subcutaneous injections for a total dose of 30 mg. It has been shown to effectively treat acute attacks in adolescent and adult patients with HAE-1 and HAE-2 [26]. Martinez-Saguer et al. [33] ecallantide (is not currently licensed in Canada but access can be requested through) the Special Access Program of Health Canada. Hypersensitivity and sometimes anaphylactic-type reactions have been described with this agent in 5% or administrations, of which approximately 50% were possible anaphylactic reactions. Subcutaneous administration is associated with fewer of these episodes (1.6%), but is still a concern [32]. In the USA, ecallantide is approved for treatment of acute attacks, but must only be administered by healthcare professionals who are trained and are prepared to treat adverse reactions.

***Recommendation 5***

**rhC1-INH is an effective therapy for the treatment of acute attacks.**

**Level of Evidence: High** (100% Agree)

**Strength of Recommendation: Strong** (97% Agree, 3% Abstain)

#### Clinical considerations

The rhC1-INH (conestat-alpha) is generated in the mammary glands of transgenic rabbits and is identical to pdC1-INH except for the degree of protein glycosylation [34]. This difference in glycosylation results in shorter plasma mean half-life of the recombinant product [33, 35], however the effect this has on physiologic activity is not known [30]. It has been shown to effectively treat acute attacks in adult patients with HAE-1 and HAE-2 [28]. It is administered intravenously at a dose of 50 U/kg in people up to 84 Kg and at a dose of 4200 U for people above 84 Kg. Because of an isolated anaphylactic reaction after administration of rhC1-INH to a rabbit allergic person, those with anti-rabbit IgE should be excluded before prescribing. It is not currently licensed in Canada but access can be requested through the Special Access Program of Health Canada.

***Recommendation 6***

**Attenuated androgens should not be used to treat acute attacks.**

**Level of Evidence: Low** (97% Agree, 3% Abstain)

**Strength of Recommendation: Strong** (100% Agree)

***Recommendation 7***

**Tranexamic acid should not be used to treat acute attacks.**

**Level of Evidence: Low** (93% Agree, 7% Disagree)

**Strength of Recommendation: Strong** (83% Agree, 10% Disagree, 7% Abstain)

#### Clinical considerations

Attenuated androgens such as the 17 α-alkylated anabolic androgen danazol and anti-fibrinolytic drugs such as tranexamic acid have not been shown to be efficacious in the treatment of acute attacks of HAE-1 and HAE-2. Given the lack of evidence for these agents in the acute treatment of HAE, the Committee strongly agreed that they should not be used for the treatment of acute HAE attacks as other agents with documented efficacy are available in Canada.

***Recommendation 8***

**Frozen plasma could be used for treatment of acute attacks if other recommended therapies are not available.**

**Level of Evidence: Low** (96% Agree, 4% Abstain)

**Strength of Recommendation: Strong** (100% Agree)

#### Clinical considerations

Frozen plasma (FP) is a blood product which contains C1-INH in association with other plasma proteins. Frozen plasma (FP) is not as safe as solvent detergent plasma (SDP) with respect to pathogen inactivation, and the level of evidence that frozen plasma is effective in the treatment of acute attacks of HAE −1 and HAE-2 is low. It also contains potential substrates for the generation of additional bradykinin and in theory could worsen attacks of angioedema. Also, not all blood banks in Canada stock FP and there are special requirements to enable access to SDP. Hence, there may be a significant delay in getting FP and/or SDP in a timely manner - in some cases up to 24 hours. Therefore, it was strongly felt by the Committee that frozen plasma products, although potentially beneficial, should only be used if other recommended therapies are not available and that every effort should be made to ensure timely and appropriate therapy for acute attacks [36, 37].

***Recommendation 9***

**We recommend early treatment of attacks to reduce morbidity (Level of Evidence: Moderate) and mortality (Level of Evidence: Expert Opinion).**

**Level of Evidence: Moderate** (92% Agree, 8% Disagree)

**Strength of Recommendation: Expert Opinion/Strong** (92% Agree, 4% Disagree, 4% Abstain)

#### Clinical considerations

Early treatment likely leads to more rapid symptom resolution. Observational studies have suggested that early treatment can be efficacious in reducing the duration of an attacking some patients [38–42]. Therefore, despite the absence of a high level of evidence, expert opinion was strong endorsing early treatment in an attempt to reduce morbidity and likely mortality. Because of the potential barriers in accessing therapy in a timely manner, patients should be trained on how to self-administer therapies appropriate for the treatment of acute attacks of HAE. If patients are not able to self-administer their own therapy, efforts should be made to ensure that this therapy is made available to them without a significant delay (see Recommendation #21).

***Recommendation 10***

**All attacks of angioedema involving the upper airway are medical emergencies and must be treated immediately. (Level of Evidence: Low) In addition, we recommend emergency department assessment. (Level of Evidence: Expert Opinion).**

**Level of Evidence: Low** (96% Agree, 4% Disagree)

**Strength of Recommendation: Expert Opinion/Strong** (100% Agree)

#### Clinical considerations

Attacks of HAE are unpredictable and potentially life-threatening. Mortality due to laryngeal angioedema is well recognized [3]. All attacks of laryngeal angioedema should be considered medical emergencies, and therapies that have been shown to be effective in the treatment of HAE should be readily available and given immediately. It is also recommended that all patients with laryngeal edema, even following self-therapy, be assessed in the emergency department in the event that the angioedema does not respond to therapy, and expertise in airway management is required [43].

### Treatment of acute attacks of HAE with normal C1 INH function

#### Background

HAE-nC1INH is a rare disease that can be a challenge to diagnose with certainty as was discussed above. It has been suggested, without confirmatory evidence that bradykinin may play a role in the pathogenesis of this disease which has led to speculation that therapies used for HAE-1 and HAE-2 may be beneficial [13]. There is also indirect evidence that anti-histamine therapy is not effective in this patient group [44]. Owing to the difficulty in identifying this subset of patients with HAE, and that there have been neither significant case series nor controlled clinical trials with respect to therapeutic intervention for acute attacks, we cannot recommend specific therapeutic interventions as this time.

***Recommendation 11***

**There is insufficient evidence to make a recommendation for or against the use of HAE-specific therapies in the treatment of acute attacks in patients with HAE with normal C1-INH.**

**Level of Evidence: Very Low** (96% Agree, 4% Disagree)

**Strength of Recommendation: Insufficient Evidence** (N/A)

#### Clinical considerations

The committee felt that there was insufficient evidence to make a specific recommendation regarding the use of therapies that have been shown to be efficacious for the acute treatment of HAE-1 and HAE-2 in patients with HAE-nC1INH.

However, in spite of this, if patients meet the clinical profile of HAE-nC1INH, a trial of HAE specific therapy could still be considered with the understanding that there is a very low level of evidence to support this and some reports demonstrate lack of efficacy for use of either pdC1INH or icatibant [45–48]. This may not be surprising given that there are neither abnormalities in C1-INH level or function, nor any confirmatory evidence if a role for bradykinin.

### Short-term prophylaxis

#### Background

STP refers to the practice of treating patients to reduce the risk of associated and consequent morbidity and mortality during a period of time when there may be an increased risk of having an attack of angioedema.

It is well recognized that physical trauma, as can occur during medical and dental procedures, can induce episodes of angioedema [49, 50]. Upper airway manipulation, including during dental surgery and intubation, is at particularly high risk due to its association with upper airway swelling. However, even minor procedures can precipitate angioedema and the ability to predict when this may occur cannot be made with certainty. Attacks can occur anywhere from hours to several days after a procedure [49].

It is also suspected that other causes, such as emotional stressors can precipitate attacks. Individual patients may also be aware of specific triggers that have been known to trigger their attacks.

Despite these observations, there is a lack of controlled clinical trials in this area, and most data come from retrospective reviews and surveys [49, 51–53].

***Recommendation 12***

**Short-term prophylaxis should be considered prior to known patient-specific triggers and for any medical, surgical or dental procedures.**

**Level of Evidence: Low**(96% Agree, 4% Disagree)

**Strength of Recommendation: Strong** (93% Agree, 7% Disagree)

***Recommendation 13***

**HAE-specific acute treatment should be available during and after any procedure.**

**Level of Evidence: Low**(92% Agree, 4% Disagree, 4% Abstain)

**Strength of Recommendation: Strong** (100% Agree)

#### Clinical considerations

There was extensive discussion as to when STP should be used and consideration was given to the development of a list of high and low risk procedures in this context. However, there is lack of data regarding the specific risk associated with each of a wide range of medical and dental procedures; it was felt that STP should be considered for all medical, surgical and dental procedures. One study assessed the risk of angioedema following surgery without prior pre-procedural prophylaxis as 5 – 30%, irrespective of type and extent of surgery [53]. Based on this, and our inability to link the risk of an attack to a specific procedure [49, 53]; it was felt that STP should at least be considered for all procedures as well as known patient-specific triggers. This recommendation was intended to remain broad in its scope as the risk of appropriate STP would likely be minimal compared to any real or perceived risk of not using STP when felt necessary. If the decision is made not to administer STP, all patients should have two acute treatment doses of appropriate therapy immediately available as per Recommendation 13. What is not known from the current data is how many patients have been denied, or have chosen not to pursue necessary procedures due to perceived risks, or not being offered STP. Ensuring access to STP may help mitigate the risk associated with procedures and enable patients to seek and receive the care they need [54].

In the absence of rigorous data on specific dosing, pre-procedural prophylaxis with pdC1-INHconcentrate is recommended however there have not been controlled dose finding studies. Response however does appear to be dose related. In one study patients had about a 30% risk with no prophylaxis, 15% risk with 500 units of pdC1-INH which was reduced to about 5% risk at 1000 units [49]. Furthermore, given that breakthrough attacks have occurred even with prophylactic pdC1-INH concentrates at 1,000 units, at least one additional treatment for acute attacks should be available. In Canada, pdC1-INHBerinert®is approved by Health Canada for acute treatment and pdC1-INH Cinryze is approved by Health Canada for long-term prophylaxis. In Europe, Cinryze is licensed to be given 1000 units within 24 hours of the procedure, or Berinert1000 units within 6 hours of an anticipated procedure.

Attenuated androgens may be considered for STP when surgery-related risk is considered low and other HAE-specific acute treatments are not immediately available. If androgens are chosen for STP, Danazol can be considered starting 5 days before the anticipated procedure or trigger and continuing 2–3 days after the anticipated trigger (Danazol 2.5 to 10 mg/kg/day, maximum 600 mg/day) [9]. Disadvantages with androgen therapy include perceived inferior efficacy to pdC1-INH concentrate and side effects such as emotional irritation and lability, menstrual disturbance, and vaginal dryness which can occur with short term use. Attenuated androgens areal so not suitable in pregnancy nor during breast feeding, and a pregnancy test should be considered before initiation of therapy with androgens. Recurrent short-term uses may be associated with similar effects seen with long-term androgen use as discussed below.

Anti-fibrinolytic agents such as tranexamic acid have been used for STP with suggested dosages of 25 mg/kg 2–3 times daily to a maximum of 3–6 g per day, 5 days before and 2–5 days after the procedure or anticipated trigger. The efficacy for prevention of attacks, however, is unknown and this agent should only be used if other therapies are not available.

### Long-term prophylaxis in HAE types 1 and 2

#### Background

LTP refers to the use of ongoing regular treatment to prevent attacks of HAE when on demand treatment does not sufficiently meet patient treatment requirements as discussed below in the Approach to Individualized Therapy section. Prophylactic therapy may be considered for patients with recurrent episodes of angioedema to reduce the frequency, duration and severity of attacks. The specifics of when to consider and when to initiate LTP are discussed below.

***Recommendation 14***

**Long-term prophylaxis may be appropriate for some patients to reduce frequency, duration and severity of attacks.**

**Level of Evidence: High** (100% Agree)

**Strength of Recommendation: Strong** (100%)

#### Clinical considerations

The aim of LTP is to reduce the frequency and/or severity of attacks of HAE and minimize the impact of HAE on their QoL so as to enable patients to live normal lives. Some patients may be candidates for long-term therapy and the benefits and risks associated with such treatments should be explored to optimize patient care. It is important to remember that no prophylactic regimen has been associated with the complete elimination of angioedema. Therefore, despite being on prophylaxis, all patients should be equipped to treat acute attacks in a manner consistent with Recommendation #1 and an acute treatment plan should be agreed between patient and physician.

***Recommendation 15***

**Attenuated androgens are effective for long-term prophylaxis in some patients.**

**Level of Evidence: Moderate** (92% Agree, 4% Disagree, 4% Abstain)

**Strength of Recommendation: Strong** (90% Agree, 6% Disagree, 4% Abstain)

#### Clinical considerations

Controlled trials and observational studies have demonstrated that treatment with 17-alpha-alkylated anabolic androgens, such as danazol, reduces the frequency and severity of HAE attacks [55–60]. Although one of the trials was a randomized controlled trial the level of evidence for the trial was not considered High as there were insufficient details on funding, sequence generation, and outcome reporting [55]. Historically, many patients have been controlled with androgen therapy and their use in some patients may be acceptable provided that the lowest effective dose is used to achieve efficacy and minimize adverse effects. Expert opinion suggests the optimal dose for danazol, to minimize adverse effects, is ≤200 mg/day [9].

Androgens can affect serum lipid levels, can be hepatotoxic resulting in hepatitis and have been associated with hepatocellular adenoma and, in very rare cases, carcinoma [58, 61, 62]. It is recommended that all patients on androgen therapy be monitored for hypertension and have a complete blood count, liver enzymes, urinalysis, serum alpha-fetoprotein, creatine phosphokinase and lipid profile performed every 6 months, and an annual liver ultrasound [15].

Virilising effects of androgen therapy can occur and include menstrual irregularities, masculinization, irreversible voice alteration, and hirsutism. Psychological side effects include emotional irritability and lability, aggressive behaviour and depression. Androgens are associated with interactions with several medications. They are contraindicated in pregnancy and during lactation, before puberty, and in patients with androgen-dependent malignancy and hepatitis [61, 62].

Patients need to be made aware of these side effects when considering and while on androgen therapy and physicians should carefully consider the risks and benefits for the particular patient.

***Recommendation 16***

**Plasma-derived C1-INH is effective for long-term prophylaxis in some patients.**

**Level of Evidence: High** (100% Agree)

**Strength of Recommendation: Strong** (100% Agree)

#### Clinical considerations

Controlled clinical trials have demonstrated that pdC1-INH used for prophylaxis in HAE-1 and HAE-2 reduces the number, duration and severity of attacks of angioedema [27, 29]. Currently, Cinryze® (Shire) is the only approved pdC1INH product for HAE prophylaxis in Canada. However, this product has not yet been distributed in Canada. The dose of pdC1INH studied was 1000 U once or twice weekly (usually every 3–4 days). No dose finding studies have been done and at 1000 units twice a week the attacks are reduced by only 50%.

Side effects reported in trials with pdC1-INH are minimal. In the trial of Cinryze® for LTP, 21 of 24 subjects (88%) had one or more adverse events, however only three adverse events (pruritus and rash, light-headedness, and fever) were classified as possibly related to the study drug. Two patients in that study demonstrated an increase in the number of attacks of HAE. A paper based on the FDA registry of drug related adverse events, reported 10 cases of severe thrombosis related to the use of Cinryze® in three years between 2008 and 2011 [63]. The reason for these thrombotic events has not been further elucidated. It has been assumed to be related to the use of central lines. However, the use of these should also be avoided due to the associated risk of serious infection [63, 64].

On-going monitoring of a patients response to therapy is recommended. In addition, as intravenous therapy requires ongoing venous access follow-up should ensure proper technique is used to maximize the health of the veins.

***Recommendation 17***

**Anti-fibrinolytics are effective for long-term prophylaxis in some patients.**

**Level of Evidence: Moderate** (96% Agree, 4% Disagree)

**Strength of Recommendation: Strong** (86% Agree, 14% Disagree)

#### Clinical considerations

The benefit of the anti-fibrinolytic agent tranexamic acid was demonstrated in a randomized placebo controlled trial with 18 subjects aged 12 years and overtaking 1 g of tranexamic acid three times a day [65], and a double-blind crossover study of epsilon amino-caproic acid in 9 patients aged 7 to 40 years resulting in these agents being given a moderate level of evidence [66]. These data suggested that anti-fibrinolytic agents could be useful for LTP for HAE-1 and HAE-2. However, their role in current LTP was felt to be justified only in some patient groups due to the lack of efficacy and the potential side effects at the dosage studied. Although not specifically studied in paediatric patients, it was felt, due to the concern of using attenuated androgens in this patient demographic, that anti-fibrinolytic agents could be considered. The recommended dosage for tranexamic acid is 30–50 mg/kg daily divided in 2 or 3 doses to a maximum of 6 g per day.

***Recommendation 18***

**It is not necessary to fail other long-term prophylaxis therapies before use of pdC1-INH for long-term prophylaxis is considered.**

**Level of Evidence: Expert Opinion** (100% Agree)

**Strength of Recommendation: Strong** (100% Agree)

#### Clinical considerations

The elements to consider when deciding to start prophylaxis are discussed below, in the approach to individualized therapy section. However, there is no recommended order or hierarchy for which therapies should be chosen for LTP. This should be based on the efficacy of the therapy, its side effects and safety, and the patient’s preference. The participants were unanimous in their recommendation that should a patient require long-term prophylaxis they can be started on prophylactic pdC1-INH without need to be tried on other prophylactic therapies first.

### Long-term prophylaxis in HAE with normal C1-INH function

#### Background

Patients with HAE-nC1INH share similar clinical characteristics with HAE-1 and HAE-2 patients, including the risk of random unpredictable attacks of debilitating and potentially life threatening angioedema [44]. These similarities have led to speculation that treatments used for LTP for HAE-1 and HAE-2 may be beneficial for patients with HAE-nC1-INH; however, due to the lack of data a recommendation for this intervention could not be made.

***Recommendation 19***

**There is insufficient evidence to make a recommendation for or against long-term prophylaxis for patients with HAE with normal C1-INH.**

**Level of Evidence: Very Low** (100% Agree)

**Strength of Recommendation: Insufficient Evidence** (N/A)

#### Clinical considerations

The absence of good evidence in the LTP of HAE with nC1-INHpatients makes it difficult to make specific recommendations regarding treatment. Patients should avoid known triggers of angioedema such as exogenous estrogen and angiotensin converting enzyme (ACE) inhibitors. There is some evidence that progesterone, anti-fibrinolytics and attenuated androgens may be efficacious in patients with HAE-nC1INH [12]. However, the data were of low quality and uniform recommendations could not be made regarding their use. The committee felt strongly that more data are needed in this area and appropriate trials should be done to help guide future treatment recommendations.

### Self-administration

#### Background

Self-administration refers to the treatment of patients outside of a health care facility either by the patient’s themselves or by a trained caregiver. The recognition and support of self-administration as treatment for HAE go back to the first international consensus document on HAE in 2003 and has been repeatedly recommended in subsequent consensus statements and guidelines [9, 15, 67]. It has been shown to be a safe and convenient option for patients, allows for early treatment, and may reduce the overall treatment costs of this group when compared to hospital-based therapy [68]. However, despite the demonstrated benefits of self-administration in terms of efficacy and improved QoL, an online survey done in the USA revealed that only 8.1% of treating physicians had patients who self-treated and only 3.5% received home healthcare assisted administration [69, 70]. Although specific data in Canada is lacking, there is little reason to think it would differ much from these findings. Self-administration of blood products for rare blood disorders is not without precedent and has been the cornerstone of effective therapy for hemophilia for more than three decades in Canada [71].

Treatment is more efficacious when attacks are treated early [72]. Evidence has shown that the earlier an attack is treated the sooner it resolves [26, 42, 73, 74]. The ability to treat an attack early depends on reducing the number of steps required between recognition of an attack that requires treatment and implementation of effective treatment. Obligating patients to travel to a health care facility to receive a therapy which has been shown to be effective when administered at home, or outside of a healthcare facility, adds to the delay in receiving treatment, may result in many attacks not being treated. Patients may also face difficulties in accessing treatment if local healthcare facilities are unfamiliar with this condition. The World Allergy Organization’s global guideline emphasizes that all therapies should be available to all HAE patients worldwide and that home- and self-administration are preferred because they reduce morbidity, absenteeism, cost, disease burden and potentially mortality, as well as improveQoL [15, 75].

Carrying a personal supply of pdC1-INH by patients has been shown to reduce the time spent waiting for treatment [5]. Additionally, patients who self-administered or had on-demand therapy have been shown to have reduced severity and duration of attacks, and an improved QoL.

***Recommendation 20***

**All patients should be trained on self-administration of HAE-specific therapies if they are suitable candidates. If patients cannot self-administer therapy, provisions should be made to ensure timely access to all appropriate therapies.**

**Level of Evidence: Low** (100% Agree)

**Strength of Recommendation: Strong** (100%)

#### Clinical considerations

Although the level of evidence was low for the recommendation that all patients should be trained on self-administration of HAE-specific therapies if they are suitable candidates, it was considered a strong recommendation unanimously. This is consistent with prior consensus statements and guidelines [9, 15, 76]. The importance of early therapy should not be underestimated, and barriers that affect its implementation should be removed. Currently in Canada, pdC1INH is licensed for on demand therapy (Berinert®) and for routine prophylaxis (Cinryze®). Because pdC1INH is a blood product and dispensed by blood banks in Canada, blood banks should adapt uniform operating procedures to enable access to pdC1INH by all suitable candidates and ensure uniform care for all patients regardless of location. The *Canada Health Act* is intended to guarantee equal access to health services and health care. Geographic disparities in care are known to exist. Self-administration of therapy in HAE will remove these disparities. Although, pdC1INH is an intravenous product and requires special considerations including product tracking and patient training, the use of intravenous blood products for self-administration is not unique. Hemophilia self-administration programs, which are similar, have been widely implemented across Canada and have been shown to be effective [71, 77].

Treatments that do not require intravenous access for either acute treatment or prophylaxis would simplify self-administered treatment. Ecallantide and icatibant are efficacious subcutaneous therapies which are administered for the treatment of acute attacks as discussed above. Ecallantide is not licensed in Canada but may be accessed through the special access program. Ecallantide should usually be administered only by a healthcare practitioner with experience and facilities to treat anaphylactic reactions which occur in 3-8% of patients. Clinical trials are also being conducted evaluating the use of subcutaneous C1INH for LTP therapy [78, 79].

Although not all patients will be suitable candidates for self-administered therapy, the option should be considered in the overall care plan of HAE patients. If patients are considered appropriate, and willing to learn self-administered therapy, they should agree to specific criteria as outlined in previously published international home-therapy guidelines [80, 81]. With self-administered therapy, patients need to be regularly monitored to ensure appropriate control of their symptoms, compliance and competency. This is discussed further in the section on Individualized therapy.

### Approach to individualized therapy

#### Background

HAE is a dynamic chronic disease and attacks of angioedema can vary in frequency and severity over the patient’s lifetime. This variability makes it important for patients to be evaluated regularly to ensure that therapy is appropriate and is being used correctly, and that side effects of therapies are being minimized. A recently published document outlines an approach to monitoring attack frequency and severity [76].

Perhaps one of the most challenging areas in patient treatment is deciding when to start or stop LTP therapy. Although guidelines exist on which agents to use when starting LTP, there is no evidence comparing the use of LTP to acute on-demand therapy regarding benefit and risk. In the absence of such evidence, given the clinical importance of this therapeutic approach, the committee attempted to determine which variables should be considered when trying to decide when to start or stop LTP.

***Recommendation 21***

**The decision to start or stop long-term prophylaxis depends on multiple factors and should be made by the patient and an HAE specialist.**

**Level of Evidence: Expert Opinion** (100% Agree)

**Strength of Recommendation: Strong** (97% Agree, 3% Disagree)

#### Clinical considerations

LTP should be considered when on-demand therapy does not allow HAE patients to lead healthy and productive. There was considerable discussion regarding factors that should be considered when deciding to start LTP. It was generally agreed that the key considerations in making the decision included the efficacy of on-demand therapy to control the severity and frequency of attacks. Although in the past some consensus documents have tried to define the number and severity of attacks as a reference point to consider when to start LTP [10], there was significant concern about the arbitrary nature by which this would be defined. This approach might lead to denying LTP to patients whose QoL is impacted, yet not meeting a specifically defined frequency of attacks. It was felt that, although the frequency of attacks is important, it is only one among many factors including severity of previous attacks, how readily patients can access emergency treatment, and their ability to administer on demand therapy, which should also be considered.

Although the aim of LTP is to reduce the number and severity of attacks, it does not eliminate the risk completely. Patients must be aware that starting LTP does not mean that they will no longer have attacks and that those attacks can still be fatal. All patients must have a plan to treat attacks on demand despite being on LTP therapy. All patients must be monitored to ensure that LTP is efficacious and that side effects are being recorded [76].

When starting LTP it is important to understand and emphasize that LTP is not necessarily a lifelong therapy and that treatment needs ongoing re-evaluation. It may be helpful to try to define what the expectations are as objectively as possible when starting LTP. Part of the monitoring process should be to examine these goals and ensure they are being met.

The decision to stop LTP also generated significant discussion. All participants felt LTP with androgens should be stopped immediately if a patient became pregnant, was breast feeding or if the patient was less than Tanner stage 5 development. Other factors that may lead to the consideration of stopping LTP is ongoing stable control with LTP therapy with no evidence of breakthrough attacks of angioedema. If the decision to stop LTP is made, all patients must ensure that they have access to the administration of appropriate on-demand therapy of acute attacks as is consistent with Recommendation #20. All members of the patients comprehensive care team should be aware of the plan to stop LTP in case complications arise.

When stopping LTP with attenuated androgens or anti-fibrinolytics, the majority of participants agreed that a gradual taper is recommended, if the patient is not pregnant, while monitoring the frequency of and the impact on the patient’s QoL. When stopping LTP with pdC1-INH it was felt it could either be stopped abruptly or the frequency of administration decreased, while monitoring the patient’s response.

The committee was unanimous that the decision to start or stop LTP should be made jointly by the patient and an HAE specialist. The patient needs to be informed of the risks and benefits of all therapies, as discussed in the relevant sections above, to enable making an informed decision. Particular attention is needed when attenuated androgens are being considered for LTP in special populations such as women of childbearing age and children. Additionally, long-term effects on vein health need to be considered when considering repeated IV infusions.

### Quality of life

#### Background

The Constitution of the World Health Organization (WHO) defines health as “A state of complete physical, mental, and social well-being not merely the absence of disease.” It follows that the measurement of health and the effects of health care must include not only changes in the frequency and severity of diseases but also an estimation of wellbeing and HRQoL. The impact of HAE on a person’s HRQoL can be considerable. A survey done in the USA in 2004 revealed that 85% of patients were afraid of sudden closure of their airway, 75% experienced intolerable pain and 53% were concerned about transmitting HAE to their offspring [82]. A recent study of 457 HAE patients from the USA reported significantly poorer health-related QoL versus population norms, based on the SF-12 Physical Component Summary and Mental Component Summary [83]. Productivity was also markedly impaired in all Work Productivity and Activity Impairment-General Health categories, including 34% overall work impairment. Because of their most recent HAE attack, workers lost a mean of 3.3 days; students lost a mean of 1.9 days. In a Swedish registry of HAE patients missed days from work and school were documented [84]. In a multicenter European Study absenteeism from work and school as well as marked loss in productivity with the most recent attack and in between attacks were recognized [85].

The Burden of Illness Study in Europe (Denmark, Germany, Spain) have shown that HAE had a high impact on daily activities during attacks and that HAE also impacted patients’ daily activities between attacks [73, 85, 86]. In this study patients also reported substantial anxiety about future attacks, traveling, and passing HAE to their children [82]. Based on Hospital Anxiety and Depression Scale scores, 38 and 14% had clinically meaningful anxiety and depression, respectively [73]. Moreover, 51% (n = 84) indicated that HAE had hindered their career/educational advancement [85].

In Sweden and France, attack frequency was shown to have a negative effect on HRQoL as measured by EQ5D today [84] or SF-36 [87]. Attack severity was shown to be related with absenteeism [84, 85].

HAE has a significant impact on QoL both during and between attacks and on absenteeism during attacks [73, 84, 85]. In aSpanish study assessing the development of a disease-specific QoL questionnaire for adult patients with hereditary angioedema due to C1 inhibitor deficiency, the factors cited most often by both experts and patients as affecting their QoL included potentially life-threatening attacks; the adverse side effects of medication (in several cases associated with chronic treatment); the unavailability of acute specific treatment at several health care centres; hereditary transmission; the lack of a known trigger which could be avoided; and the fact that it is a rare disease about which health care professionals know very little. The authors particularly noted that patients and experts may not agree on what are the most relevant aspects of HAE [88]. Aesthetics was mentioned more often by patients than by experts. On the other hand, experts were more likely to mention the adverse side effects of treatment. This finding supports the general opinion that the clinician’s view of disease severity does not necessarily match with the patient’s perception.

***Recommendation 22***

**Health care providers should specifically address factors known to affect quality of life with HAE patients. Management of HAE should aim to improve patients’ quality of life.**

**Level of Evidence: Low** (100% Agree)

**Strength of Recommendation: Strong** (100% Agree)

#### Clinical considerations

Assessment of HAE control as it relates to the frequency, duration and severity of attacks is not the only thing to consider when monitoring patients. Data suggests that factors which relate to a patients qualify of life are important when following patients with HAE. An international specific HRQoL questionnaire for adult patients with HAE due to C1-inhibitor deficiency has been developed [89] and is available for its use in clinical practice. The factors that impact a patient’s QoL may be different than those anticipated by the healthcare providers. Modifications should be made to improve a patient’s QoL wherever possible. A small study demonstrated that self-administration of pdC1-INH improved QOL on both physical and psychological parameters. Patients were able to resume a normal life without restriction [75]. Implementation of self-administered therapy may lead to an improved QoL by reducing the suffering caused by treating attacks too late or leaving them untreated altogether. It was reinforced at the meeting by the patient representatives that there are still significant barriers to getting timely and appropriate therapy at centres across Canada, likely from the lack of awareness of HAE and the appropriate therapies available for treatment. On-going research is required to determine additional factors that may have impact HAE patients’ QoL.

### Comprehensive care

#### Background

Comprehensive care of patientsis based on integration of the organization, delivery, and management of services related to diagnosis, treatment, care, rehabilitation and health promotion. The comprehensive care model has been adopted by many rare disease groups and there is evidence in other rare diseases that this model results in better patient outcomes and reduced costs [71]. Haemophilia has used this model for decades. HAE is similar to other rare blood disorders, including haemophilia, because it is a chronic condition that is potentially life threatening and requires a highly specialized, multidisciplinary team to manage. However, although HAE is similar to other conditions, it is also different enough to require its own framework to meet the specific needs of these patients. The recommendation to provide comprehensive care for patients with HAE is not new and exists in previously published guidelines. The specific elements of comprehensive care for HAE in Canada were published previously and are listed in Table 4.

**Table 4:** **Requirements for comprehensive care in the management of hereditary angioedema patients**

**Best Clinical Treatment outcomes including:**	
a.	A comprehensive care team made up of nurse coordinator, clinician, social worker, data manager, pain management specialist, genetic counsellor, and administrative support;
b.	Access to specialized diagnostic testing;
c.	Access to home treatment;
d.	A networked Patient Information System to facilitate product recalls - collect data on therapy outcome measures and safety, and facilitate participation in clinical trials
e.	Access to clinical advances as they become available;
f.	Access to 24 hour support;
g.	Access to up-to-date standards of care, including standardized wallet cards;
h.	Tracking and intermittent audit of quality outcomes including beneficial and adverse outcomes through secure, comprehensive and networked data management.
**Education of patients and staff regarding:**	
a.	Responsible Self/Family Care (home care model) with home and self-infusion/administration instruction and support;
b.	Developments in the cause, diagnosis, treatment, outcomes, and prognosis of HAE
c.	Changes in the administrative management of the clinic
**An environment conducive to research including:**	
a.	Access to and support for clinical trials of new treatments;
b.	Access to and support for translational research in diagnosis and prognosis;
c.	Access to and support for psychosocial research such as quality of life studies.
**An advisory or oversight board with patient group representation for each clinic**	

Reference [9].

***Recommendation 23***

**Comprehensive care should be available for all patients with HAE.**

**Level of Evidence: Low** (100% Agree)

**Strength of Recommendation: Strong** (100% Agree)

#### Clinical Considerations

Although the importance of the comprehensive care model in HAE was recognized by the committee unanimously, and specific recommendations have existed with respect to its requirements, this care model is not available to all patients with HAE in Canada. The provincial and territorial model of health care funding makes implementation of nationally uniform HAE comprehensive care clinics challenging. Despite this, the fundamentals of comprehensive care should be uniform across the country and equally accessible across all geographic locations. Support should be provided by provincial and territorial governments to ensure that proper standards of care are being met. Treatments for HAE can be expensive; however inappropriate treatment of HAE may be even more costly. It was recognised by the committee that on-going monitoring of comprehensive care programs is essential to measure their impact on patients’ outcomes such as disease control, QoL and economic effects.

## Special populations

The committee recognized that management of HAE in certain populations were not specifically addressed in this guideline. These include paediatric HAE patients and HAE patients during lactation and menstruation. The recently published WAO document specifically addressed recommendations for the management of HAE-1 and −2 in children [15]. An international consensus and practice guidelines on the gynaecologic and obstetrical management of female patients with hereditary angioedema caused by C1 inhibitor deficiency was recently published by Caballero et al. [90] and readers are referred to those publications with the understanding that they are not specific to Canadians.

## Electronic supplementary material

Additional file 1: **Conflict of Interest Form.** (PDF 7 MB)

Additional file 2: **Search Strategy.** (DOCX 17 KB)

Additional file 3: **Levels of Evidence and Strength of Recommendation.** (DOCX 19 KB)

Additional file 4: **HAE RCT Evidence Tables.** Includes data extraction, quality assessments, and study reference codes and citations for randomized control trials. (XLSX 28 KB)

Additional file 5: **HAE Lower Quality Comparison Study Evidence Tables.** Includes data extraction, quality assessments, and study reference codes and citations for lower quality comparison studies. (XLSX 52 KB)

## Abbreviations

HAE: Hereditary Angioedema
C1-INH: C1 inhibitor
C1-INH-HAE: Hereditary Angioedema due to C1Inhibitor Deficiency
HRQoL: Health Related Quality of Life
HAE-1: Hereditary Angioedema Type 1
HAE-2: Hereditary Angioedema Type 2
HAE-nC1INH: Hereditary Angioedema with Normal C1 Inhibitor
*F*12: Factor 12
HAE-nC1INH-FXII: Hereditary Angioedema due to Factor XII mutations
HAE-nC1INH-unknown: Hereditary Angioedema due to unknown cause
QoL: Quality of Life
STP: Short-term Prophylaxis
LTP: Long-term Prophylaxis
CHAEN: Canadian Hereditary Angioedema Network
RCAH: Réseau Canadien d'angioédème héréditaire
pdC1-INH: Plasma Derived C1-inhibitor
rhC1-INH: recombinant C1-inhibitor.
