# Supplementary material for: Canadian hereditary angioedema guideline
Source: Allergy Asthma Clin Immunol. 2014 Oct 24;10(1):50. doi: 10.1186/1710-1492-10-50 (PMC4210625; doi:10.1186/1710-1492-10-50)
Supplement: Supplementary file 2 — Additional file 2: Search Strategy. (DOCX 17 KB) [file 13223_2014_521_MOESM2_ESM.docx]

## Appendix 2: Search Strategy

### Search Strategy

Database: Ovid MEDLINE(R) <1946 to October Week 1 2013>

Search conducted on October 10, 2013 by Kelly Lang-Robertson, MLIS

1 angioedemas, hereditary/ or hereditary angioedema type iii/ or "hereditary angioedema types i and ii"/ (473)

2 ("hereditary angio$" or "inherited angio$" or "familial angio$").mp. (1621)

3 hereditary C1-INH deficiency.mp. [mp=title, abstract, original title, name of substance word, subject heading word, keyword heading word, protocol supplementary concept, rare disease supplementary concept, unique identifier] (17)

4 or/1-3 (1682)

5 ("clinical trial" or random$ or placebo).mp.ortu.xs. (4184453)

6 4 and 5 (815)

7 limit 4 to (clinical trial, all or clinical trial, phase i or clinical trial, phase ii or clinical trial, phase iii or clinical trial, phase iv or clinical trial or comparative study or controlled clinical trial or randomized controlled trial) (126)

8 6 or 7 (850)

9 limit 8 to (english language and humans) (692)

10 limit 9 to (case reports or comment or editorial or letter or news) (256)

11 9 not 10 (436)

*(Note: 20 of the results from the final set were identified in the abstract review stage as duplicates)*

### PICO

**Population:**

- Patients diagnosed with type I or type II hereditary angioedema
- Patients diagnosed with type III hereditary angioedema (HAE with Normal C1-INH Function

**Intervention:**

**Acute Treatment**

- C1-INH
- rhC1-INH (Ruconest)
- Kallikrein inhibitors (Ecallantide)
- Bradykinin receptor antagonists (Icatibant)
- Antifibrinolytic drugs (tranexamic acid)
- Solvent/detergent-treated plasma (SDP)
- Fresh frozen plasma (FFP)

**Long-Term Prophylaxis**

- C1-INH
- Attenuated androgens (Danazol)
- Antifibrinolytics (tranexamic acid)
- rhC1-INH (Ruconest)
- Synthetic steroids (Tibolone)
- E-aminocaproic acid (EACA)

**Short-Term Prophylaxis**

- C1-INH-NF (Cinryze)
- Attenuated androgens (Danazol)
- Antifibrinolytics (tranexamic acid)
- Anabolic steroids
- Fresh frozen plasma (FFP)

**Comparison:**

- Any, including:
  - Placebo
  - Regular treatment
  - No intervention

**Outcome:**

- Frequency or severity of attacks, or symptom relief including:
  - Time to symptom relief (onset or complete resolution)
  - Mean symptom complex severity score (MSCS score)
  - Treatment outcome score (TOS)
  - Attack duration
  - Time to treatment
  - Rebound/relapse
  - Number of attacks (eg. median per year or per month)
  - Overall QoL
