# Supplementary material for: Canadian hereditary angioedema guideline
Source: Allergy Asthma Clin Immunol. 2014 Oct 24;10(1):50. doi: 10.1186/1710-1492-10-50 (PMC4210625; doi:10.1186/1710-1492-10-50)
Supplement: Supplementary file 3 — Additional file 3: Levels of Evidence and Strength of Recommendation. (DOCX 19 KB) [file 13223_2014_521_MOESM3_ESM.docx]

## Appendix 3: Levels of Evidence and Strength of Recommendation

Note: Levels of Evidence and Strength of Recommendation were adapted from GRADE ^1,2,3^

### Table 1: Levels of Evidence

| **Quality Level** | **Definition** |
| --- | --- |
| High | We are very confident that the true effect lies close to that of the estimate of the effect |
| Moderate | We are moderately confident in the effect estimate: The true effect is likely to be close to the estimate of the effect, but there is a possibility that it is substantially different |
| Low | Our confidence in the effect estimate is limited: The true effect may be substantially different from the estimate of the effect |
| Very Low | We have very little confidence in the effect estimate: The true effect is likely to be substantially different from the estimate of effect |
| Note: If no published evidence was identified in an area, but the Committee and Conference Participants determined that it was important to make a recommendation this was labeled as Expert Opinion. | |

### Determining Levels of Evidence

All non-randomized, non-blinded trials were considered to be Low Level of Evidence.

All randomized control trials were considered a High Level of Evidence, and were downgraded based on the following parameters:

| **Parameters** | **Examples of Limitations** | **Effect on Level of Evidence *** |
| --- | --- | --- |
| Limitations of Design / Risk of Bias  (Cochrane Risk of Bias tool) | - Lack of allocation concealment - Lack of adequate sequence generation - Lack of blinding - Incomplete data - Selective reporting - Other limitations such as stopping early | Majority of items not satisfied or not reported = downgraded by 1 level |
| Inconsistency | - Large variation in effect - Poor heterogeneity of results | Downgraded by 1 level |
| Imprecision of Results | - Small sample size - Wide confidence intervals around the estimate of the effect - Study is underpowered | Noted but not downgraded |
| Publication Bias | - Bias introduced due to significant industry funding | Noted but not downgraded (majority of studies were industry funded) |
| Indirectness / Generalizability | - Study population or setting differs significantly from population of interest | Not applicable |

*If it was determined that the limitation was significant, the Level of Evidence was downgraded by 2 levels.

### Table 2: Strength of Recommendation

| **Recommendations can be either STRONG or WEAK**  **Strength of Recommendation is Determined By:**   1. Quality of Evidence  - The higher the quality of evidence, the higher the likelihood that a strong recommendation is warranted  1. Balance Between Desirable and Undesirable Effects  - The larger the difference between the desirable and undesirable effects, the higher the likelihood that a strong recommendation is warranted.  1. Values and Preferences  - The more values and preferences vary, or the greater the uncertainty in values and preferences, the higher the likelihood that a weak recommendation is warranted  1. Costs (Resource Allocation)  - The higher the costs of an intervention—that is, the greater the resources consumed—the lower the likelihood that a strong recommendation is warranted |
| --- |

### Factors considered when deciding on a strong or weak recommendation

- Uncertainty in the estimates of likely benefit, and likely risk, inconvenience, and costs
- Importance of the outcome that treatment prevents
- Magnitude of treatment effect
- Precision of estimate of treatment effect
- Risks associated with therapy
- Burdens of therapy
- Risk of target event
- Costs
- Varying values

### The implications of a strong recommendation are

- Clinicians: Most patients should receive the recommended course of action
- Patients: Most people would want the recommended course of action and only a small proportion would not
- Policy makers: The recommendation can be adopted as a policy in most situations

### The implications of a weak recommendation are

- Clinicians: Different choices will be appropriate for different patients and that you must help each patient to arrive at a management decision consistent with her or his values and preferences
- Patients: Most would want the recommended course of action, but many would not
- Policy makers: Policy making will require substantial debate and involvement of many stakeholders
